# Vocational Rehabilitation for Patients with Chronic Musculoskeletal Pain With or Without a Work Module: An Economic Evaluation

**DOI:** 10.1007/s10926-020-09921-y

**Published:** 2020-08-20

**Authors:** Michiel F. Reneman, Timo T. Beemster, Sybren J. Welling, Jochen O. Mierau, Hermien H. Dijk

**Affiliations:** 1 Department of Rehabilitation Medicine, Center for Rehabilitation, University Medical Center Groningen, University of Groningen, Haren, P.O. Box 30.002, 9750 RA Groningen, The Netherlands; 2 Department of Research and Development, Heliomare Rehabilitation Center, Wijk aan Zee, The Netherlands; 3 Department Coronel Institute of Occupational Health, Amsterdam UMC, University of Amsterdam, Amsterdam, The Netherlands; 4 Faculty of Economics and Business, University of Groningen, Groningen, The Netherlands; 5 Aletta Jacobs School of Public Health, University of Groningen, Groningen, The Netherlands

**Keywords:** Cost-effectiveness, Return on investment, QALY, ICER, CEAC, Chronic pain

## Abstract

*Purpose* Vocational rehabilitation (VR) is a widely used intervention aimed to optimize work participation for patients on sick leave due to chronic musculoskeletal pain (CMP). Economic evaluations of care as usual VR are scarce, and may provide relevant information to guide clinical, reimbursement and policy decisions. The aim of this study was to evaluate the short-term cost-effectiveness and return on investment (ROI) of VR for patients on sick leave due to CMP with an additional work module (VR+) compared to VR without work module, from a societal and employers’ perspective. *Methods* A retrospective longitudinal cohort study within a Dutch care as usual context was applied. Participants with CMP and decreased work participation originating from seven Dutch rehabilitation centers were included in this study. Participants underwent VR or VR+. Main data sources at baseline and discharge: Quality-adjusted life year (QALY) based on EQ-5D, intervention costs, self-reported productivity and health care utilization. *Main analyses* cost-effectiveness, including incremental cost-effectiveness ratio (ICER) and a cost-effectiveness acceptability curve (CEAC); and ROI analyses with use of the human capital method. *Results* N = 324 participants were analyzed. The results show that VR+ was cost-effective compared to VR: mean cost savings of €820 per 0.012 QALY gained. CEAC suggests probability of VR+ being cost-effective is > 0.91 for thresholds of €20.000 and higher. The mean ROI of VR+ for employers was 38%. *Conclusion* It was concluded that at discharge, VR+ was cost-effective compared to VR. ROI was positive for employers.

## Introduction

Chronic musculoskeletal pain (CMP) is one of the most frequent causes of work disability in the Netherlands [1]. Work disability places a large burden on both patients and on the Dutch economy through medical costs and work productivity costs which arise from impaired work participation: absenteeism and presenteeism [2–4]. Moreover, the economic burden of CMP in The Netherlands was estimated at €3.5 billion in 2007, and is expected to increase as the number of patients with CMP is expected to rise [5]. These numbers are not unique for the Netherlands; similar prevalence and impact are reported worldwide [6]. For patients, employers, health insurers, and the government it is worthwhile to seek for possibilities to reduce these costs.

For a subgroup of patients with CMP and reduced work participation, Vocational Rehabilitation (VR) may be a feasible and effective option [6–9]. VR is an interdisciplinary, multi-domain intervention, provided by a multidisciplinary team, collaborating with patients using a shared biopsychosocial model [10–14] and shared goals [15]. The primary aim of VR is to optimize work participation [16]. Effectiveness of VR might be higher when workplace involvement is added (e.g. case management, workplace visit, consultations) [6, 8, 9, 17, 18]. The extent to which workplace involvement is needed to achieve or improve effectiveness is unknown [17], and addition of a supplementary work module to a rehabilitation program has shown mixed results [19, 20]. In addition, the cost-effectiveness of a supplementary work module is unknown [6]. Within the Dutch health care system, the supplementary work module is currently not reimbursed by the government or insurance but paid by employers. It is uncertain whether the work module generates a positive return on investment for employers.

The main objective of this study was to investigate whether a supplementary work module to VR (denoted as VR+) in patients with CMP is cost-effective compared to VR, from the perspective of employers and society. The first study question was: is VR+ more cost-effective than VR? Based on current evidence, we hypothesized that VR+ was more cost-effective compared to VR. The second aim was to study the return on investment from the perspective of the employer. The clinical effectiveness of VR and VR+ was studied in a previous study, demonstrating higher odds of VR+ on work participation at discharge and 6 months follow-up [21]. The present study builds on this study, analyzing the short-term results at discharge from an economic perspective. It is based on real-world data derived from usual care, thereby filling an identified gap in knowledge [22].

## Methods

We used the Consolidated Health Economic Evaluation Reporting Standards (CHEERS) Statement as a reporting guideline [23]. The Medical Ethical Committee of the Academic Medical Center, Amsterdam, the Netherlands provided a waiver, stating that formal ethical approval was not needed within the Dutch context (number W18_194).

### Target Population and Subgroups

Working age patients (18–65 years) with subacute or chronic musculoskeletal pain and reduced work participation (full or part-time sick leave) who underwent VR or VR+ between September 2014 and July 2018 were included. Patients were not included when they had no paid work, were unable to complete questionnaires in Dutch, or did not sign informed consent [21].

### Setting and Location

This retrospective cohort study was carried out in seven outpatient rehabilitation centers in the Netherlands. Routinely collected data as part of care as usual was used. In the Netherlands, during the first 2 years of absence from work, the employee and employer are both responsible for return to work. According to the Dutch Gatekeeper Improvement Act, the employer must provide wage replacement and modified work during this 2-year period [24]. Within the Dutch health care system, VR is reimbursed. A supplementary work module is not considered ‘healthcare’ and, consequently, not reimbursed by the health care insurer. The work module (€1250) is reimbursed by the employer. All patients were offered VR+, however, patients’ whose employers refused to reimburse the work module received VR only.

### Comparators

VR is a 15-week interdisciplinary biopsychosocial group-based outpatient program, delivered by health care professionals (physiotherapist, psychologist) twice weekly, containing four contact hours per session, which amounts to ~ 90 contact hours in total. Detailed content has been described elsewhere [25]. VR consisted of multi-components from the health-focused domain. They included general exercise therapy based on principles of graded activity, CBT, group education, and relaxation. The VR+ program was the same as VR, but was extended with a work module. The work module is delivered in addition to VR by a return to work (RTW) coordinator. The work module consists of case management, the development of a RTW plan, and a workplace visit. The workplace visit consists of an at-work communication between the sick employee, employer/supervisor, and the RTW coordinator, and contains topics such as resolving barriers, discussion of the RTW plan, and possible advice for work accommodations. The work module amounts approximately 10 contact hours [25]. VR+ contains a total of ~ 100 contact hours.

### Time Horizon

Patients completed online delivered questionnaires at baseline (T0) and 14 weeks later (1 week before discharge; T1). This treatment period is equal for VR and VR+. Data was collected between September 2014 and July 2018.

### Costs

Costs were related to health care consumption and work participation (costs that arise from sick leave days (absenteeism) and productivity losses (presenteeism)).

The costs of the VR program of €5000 were paid by the health care insurer. The work module of €1250 was paid by the employer.

Health care consumption was assessed with the Trimbos iMTA (institute for Medical Technology Assessment) questionnaire for measuring Costs of Psychiatric Illnesses, VR version (TiCP-VR) [26]. TiCP-VR showed sufficient retest reliability and agreement in assessing total healthcare consumptions in sick-listed patients with CMP after attending a VR program in the Netherlands [26]. Medical costs were constructed by multiplying the utilization of health care by its reference price in €2015, provided by the Dutch Institute of Health Care [27]. This guideline for economic evaluations in health care provides average cost prices for health care treatments in The Netherlands. The price multiplied by the number of consultations sum to an aggregate medical consumption amount.

Work participation was assessed with the iMTA Productivity Cost Questionnaire, VR version (iPCQ-VR) [26]. Absenteeism was assessed as the number of sick leave days in the last 4 weeks. Presenteeism was assessed as the number of days less productive at work due to health complaints) and the presenteeism score on a 0–10 scale (0: ‘I couldn’t do anything’, 10: ‘I could do the same as normal’). Presenteeism was assessed with 4 weeks recall. Absenteeism and presenteeism items of the iPCQ-VR showed poor to moderate retest reliability and agreement in sick-listed patients with CMP after attending a VR program in the Netherlands [26]. The human capital approach was used in this study for calculating presenteeism, which takes the patient’s perspective and counts every hour not worked as an hour lost. Absenteeism and presenteeism variables were multiplied by the productivity value; the average hourly wage in the Netherlands amounts to €31.60 for women and €37.90 for men [27]. To calculate the presenteeism costs, the costs of productivity losses were multiplied by the number of workdays lost [28]. The formula for presenteeism is: Number of working days less productive * [1 − (presenteeism score/10)] * number of hours per working day [28].^1^ All calculated costs in this paper were indexed for the year 2015.

#### Effectiveness

Effectiveness was assessed with the EuroQol-5D (EQ-5D). The EQ-5D measures five dimensions: mobility, self-care, activities of daily life, pain and anxiety/depression on a categorical scale (1 to 3). A Dutch language version of the EQ-5D was used [29, 30]. The EQ-5D is a widely employed instrument used to assess health-related quality of life (QoL), and is recommended by the Dutch guideline for health economic evaluations. Quality-adjusted life years (QALYs) were calculated in three steps. First, the EQ-5D scores were converted to utility scores using the Dutch EQ-5D tariff. Second, QALYs were calculated per time period. Third, one summated QALY was calculated from the calculated QALYs in step two.

#### Sample Characteristics

The following demographic, pain-related, and work-related characteristics were collected.

### Demographic Variables: Age, Gender, and Education

Pain-related characteristics: duration of pain, number of pain locations, and pain intensity score. Pain intensity score was assessed on a 11-point Likert scale as the mean pain score in the preceding week, where 0 denoted no pain and 10 denoted worst possible pain.

Disability was assessed with the Pain Disability Index (PDI) [31, 32]. The PDI consists of 7 items, each scored from 0 to 10; a score of 0 indicates no disability and 10 maximum disability. Total score ranges 0–70.

Work ability was assessed by a single item of the Work Ability Index (WAI) [33]. It measures the current work ability compared to lifetime’s best work ability on a 0–10 response scale, where 0 represents completely unable to work and 10 represents work ability at its best [33, 34].

RTW expectation was assessed on a 0–10 scale, with patients rating the certainty that they will be working in six months, where 0 represents ‘Not at all certain’ to 10 ‘Extremely certain’ [21].

### Analytical Methods

Missing values of TICP-VR or MPCQ were replaced by 0 in categories that represent low monetary value items (example: general practitioner visit) if at least one question was answered in that category by the individual. If more than 1 or one of these low value items were missing in their entirety, we recoded this as missing of a full category (medical costs, productivity costs). If a full category was missing, total costs could not be calculated; coded as missing.

**Difference-in-Difference Estimations.** Because selection into VR and VR+ was not random but determined by employers’ willingness to pay for the work module, the results in this study might suffer from selection bias. To control for this, difference-in-difference estimations [35] were performed to assess the effect of the work module on both costs and effects. Difference-in-difference estimation considers that treatment and control group might differ at baseline, due to either observed or unobserved characteristics, and assumes that, after accounting for control variables, both groups share a common trend over time in costs and effects. Control variables included in the estimations were age, gender, education, and return to work expectation. Difference-in-difference estimations were applied in cost-effectiveness analyses and return on investment analyses.

**Interpolation.** The cost surveys were designed with 1 month to re-call, leaving a gap of 10 weeks without work productivity data between T0 and T1. Linear interpolation was used to extrapolate the difference-in-difference results to the 14-week period, assuming the effect of the intervention on both costs and QALYs to be zero at T0.

#### Cost-Effectiveness Analysis

To assess the cost-effectiveness of VR+ versus VR, the incremental cost-effectiveness ratio (ICER) provides guidance on whether the effects are worth the costs. Quality adjusted life years (QALYs) were used as an effect parameter. QALYs were calculated from the EQ-5D score following the method of Prieto and Sacristán [36]. Because QALYs are measured in years, the number of QALYs gained was calculated by multiplying the 14-week period with the change in quality of life (QOL) and dividing by 52 weeks. The costs in this section were evaluated from a societal perspective and include the work productivity, health care consumption, and intervention costs (€5000 for VR and €6250 for VR+). The difference in the total costs between T0 and T1 was executed to calculate the ICER to compare both treatments. Nonparametric bootstrapping was used to randomly replicate the sample to estimate p-values and confidence intervals, and to construct the cost-effectiveness acceptability curve (CEAC). After bootstrapping the sample observations 1000 times, the ICERs were graphed in a cost-effectiveness plane. Regression analyses for medical, productivity and total costs and EQ-5D, used as basis for ICER calculations, were calculated. To assess whether the extra QOL is worth the additional costs, a CEAC was created. The y-axis describes the probability that the work module is cost-effective against the willingness to pay per QOL on the x-axis using a nonparametric approach [37].

#### Return on Investment Analysis

The monetary value of VR+ from the perspective of employers was calculated using a return on investment (ROI) metric. Only productivity expenditures and the direct cost of the work module to the employer were now considered costs. If a patient becomes more productive after VR or VR+, the work productivity costs decline, which was considered a benefit to the employer. The ROI was calculated at discharge (T1). ROI was expressed in percentages: $ROI = \frac{{\left( {Benefits - Costs} \right)}}{Costs}\left[ {*100\% } \right]$.

## Results

The initial study sample consisted of a total of 1272 patients. Of these, n = 134 did not receive VR or VR+; they were excluded for analyses, leaving a baseline sample of n = 1038. The dataset contained a substantial number of missing values. The effect parameter EQ-5D was measured for n = 1261 patients at T0, n = 562 at T1. The medical costs contain n = 1140 observations at T0 and n = 487 at T1. Table 1 shows the descriptive statistics at baseline of the study sample with complete datasets needed to perform ICER analyses (n = 324).

**Table 1:** Baseline demographic and clinical characteristics of the study population (n = 324)

	VR (n = 88)	VR+ (n = 236)
	Mean (SD) or %	Mean (SD) or %
Age (years), mean	46.4 (10.9)	46.2 (10.7)
Gender (% female)	51	63
Education		
Low	39	18
Medium	38	49
High	24	33
Contract (hours/week)	30.6 (10.2)	31.1 (8.8)
Working days/week	4.2 (1.1)	4.1 (1.0)
Work status		
Full at work	18	8
Part-time sick leave	50	50
Full sick leave	32	42
Sick leave > 4 weeks (% yes)	42	56
Presenteeism (%yes)	69	61
Duration of complaints		
< 6 months	23	27
0.5–1 year	19	25
> 1 year	58	49
Pain intensity (0–10)	5.6 (2.1)	5.2 (2.3)
Disability (PDI 0–70)	33.8 (12.0)	34.8 (12.5)
Presenteeism (0–10)	5.0 (2.4)	5.1 (2.4)
Work ability (0–10)	3.8 (2.4)	3.8 (2.4)
RTW expectancy (0–10)	5.7 (3.2)	6.6 (2.8)

*PDI* pain disability index, *RTW* return to work

The main study parameters that formed the basis for the CEA and ROI analyses are provided in Table 2.

**Table 2:** EQ-5D, medical and work productivity costs at T0 and T1 for VR and VR+ (mean (sd) € per patient)

	VR		VR+	
	T0	T1	T0	T1
EQ-5D	0.62 (0.25)	0.66 (0.25)	0.62 (0.24)	0.76 (0.19)
Program costs HC insurance	5000	0	5000	0
Program costs employer	0	0	1250	0
Medical costs	435 (277)	325 (352)	560 (369)	291 (315)
Work productivity costs	3006 (2303)	1888 (2559)	3164 (2266)	1196 (1658)
Sum costs (society)	3440 (2376)	2213 (2598)	3724 (2414)	1487 (1773)

### Cost Effectiveness

Regression results for medical, work productivity, total costs and EQ-5D used as basis for ICER calculations are presented in Table 3, showing differences in costs of VR+ compared to VR at T1. Diff-in-diff regression coefficients at group level indicate significant decrease in costs and increase in EQ-5D. Costs of VR (€5000 medical) and VR+ (€5000 medical + €1250 work module) were not included in these analyses (but were included in the ICER and ROI analyses).

**Table 3:** Diff-in-diff regression results VR+

	VR+ compared to VR
Medical costs (€)	− 169** (57)
Work productivity costs (€)	− 845** (323)
Total costs (€)	− 1014** (337)
EQ-5D	0.074* (0.032)

Robust standard errors are in brackets

**p < 0.01, *p < 0.05

**Fig. 1:**
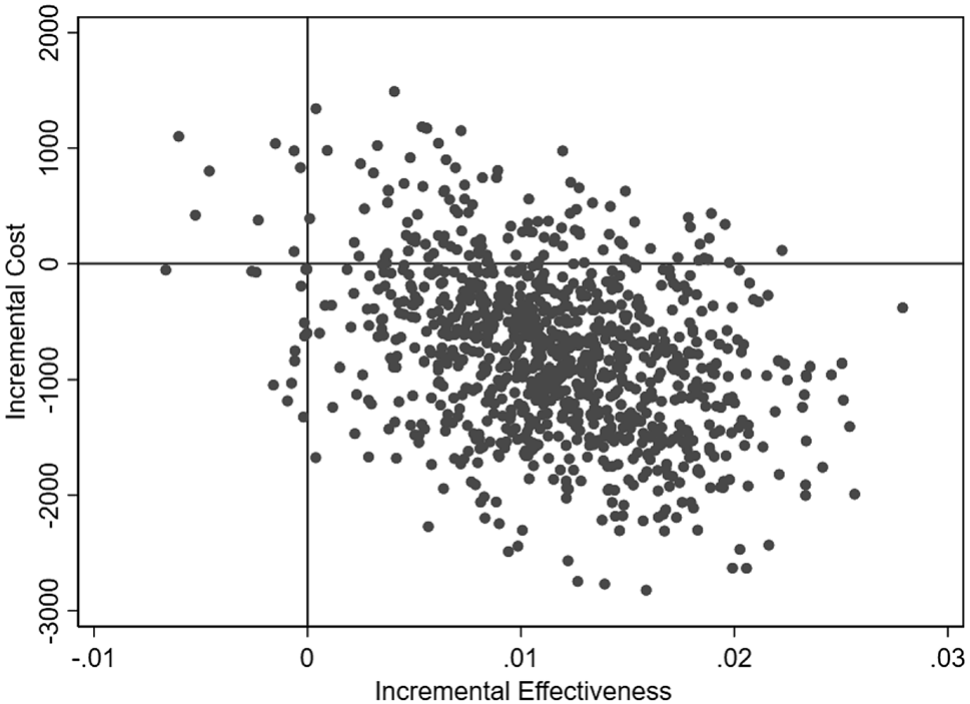
CE-plane of costs (€) and effectiveness (QALYs) of VR+ compared to VR

Results of ICER analyses are presented in a CE-plane (Fig. 1). Most of the bootstrapped replications of the QOL effects (p < 0.05) are positive and show cost-savings (p = 0.24) and are thus located in the south-eastern quadrant of the figure, indicating that VR+ is less costly and more effective than VR for most replications. The non-bootstrapped replication equals the mean costs and effects of the sample, indicating mean cost savings of VR+ of €820 per 0.0115 QALY gained over a 14-week period (P = 0.26, 95% CI from − 2.84 × 10^5^ to 1.70 × 10^5^). Mean VR+ cost savings per QALY are €71.088.

**Fig. 2:**
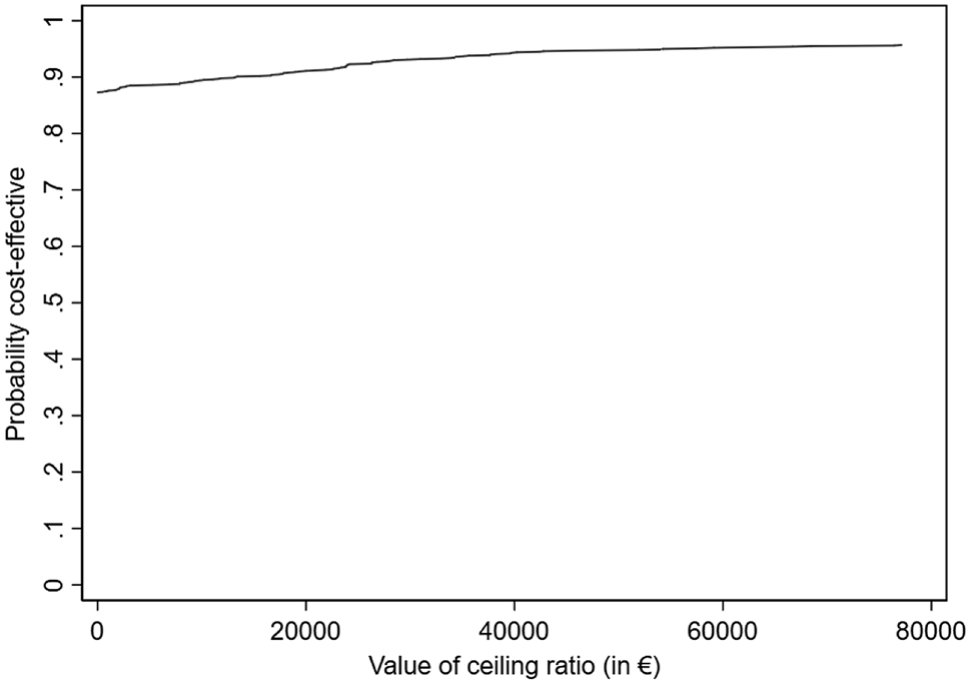
Cost-effectiveness acceptability curve indicating the probability of acceptable costs (y-axis) at a given threshold (x-axis)

The CEAC is presented in Fig. 2. The CEAC evaluates the cost-effectiveness plane for the different quality of life thresholds, indicating the willingness-to-pay from a societal perspective. Because the monetary value per QALY remains debatable and there is no consented threshold to assess cost-effectiveness, 3 thresholds are presented, including the. suggested threshold of €80.000 by the Netherlands Council for Public Health and Health Care (https://www.raadrvs.nl/documenten/publicaties/2007/10/17/rechtvaardige-en-duurzame-zorg; 2007). When applying the €80.000 threshold, the CEAC suggests a probability of 96% that costs for VR+ are acceptable. The probabilities for thresholds of €20.000 and €50.000 are 91% and 95%, respectively.

### Return on Investment

Employers invested €1250 in VR+. This resulted in higher productivity representing a mean benefit of €1725), thus a mean net benefit of €475 per participant. This represents a ROI of 38% (95% CI from − 66 to 142%). For these ROI analyses, an extra of n = 107 complete datasets were available (total n = 431). The ROI was lower (29%, 95% CI from − 72 to 129%)), but still within the 95% CI.

## Discussion

This short-term retrospective longitudinal cohort study carried out within a Dutch care as usual context suggest that VR+ was cost-effective compared to VR: ICER analyses suggest mean cost savings of €820 per 0.012 QALY gained. CEAC suggests probability of VR+ being cost-effective is > 0.91 for thresholds of €20,000 and higher. ROI of VR+ for employers 38%.

Economic evaluations of VR/VR+ have been performed alongside randomized controlled trials, and have resulted in positive outcomes [38–41], but this is among the first economic evaluations performed within usual care. While the results of the present study are in line with RCTs, adding to the robustness of the VR/VR+ knowledge base, it also fills the research gap of economic evaluations performed within usual care in this field [22, 42]. While there are advantages to evaluations using real life data, the main disadvantages of its uncontrolled design are selection bias and loss of follow-up data. Our analytical strategy, especially applying difference-in-difference analyses, controlled for baseline differences between VR and VR+ groups. By controlling for known and unknown confounders, we have limited the possibilities of bias due to baseline differences in our results and conclusions. However, these difference-in-difference estimations do rely on the assumption of a parallel trend, after accounting for control variables, for treatment and control group if the intervention had not occurred, which could not be tested due to lack of data before T0. In a different study, we have demonstrated that missing data were random, implying that this did also not introduce systematic bias [21].

As demonstrated in systematic reviews, the effectiveness of VR has been demonstrated in multiple settings [6, 8, 9, 17]. These reviews contain studies that were conducted with VR programs with different content and dosage and were performed in different jurisdictions with different healthcare and social security systems. Within the Dutch jurisdiction, the work module needed to be reimbursed by the employer. Until now, this involved an investment decision with unknown monetary benefits. As payment schemes differ between jurisdictions, it is unknown whether the figures of this study are generalizable to other systems. While in general VR in patients with CMP has a positive economic picture, detailed analyses do differ between systems. This evaluation was based on VR medical costs of €5000, based on a dosage of 90 h. There are many studies suggesting that this dosage can be lowered without loss of effectiveness [6–8, 43]. This would lead to lower medical costs and, consequently, a more positive economic picture. In the present study, presenteeism was accounted for, using self-reported productivity as a basis. Because productivity costs were a relevant part of the analyses, this has relevantly influenced our results. A gold-standard means of measuring and calculating presenteeism, however, is absent [42, 44]. A different measure may have led to different results. Even though presenteeism is considered a relevant cost-driver and it is suggested to be included in economic analyses [27], it may not be applied in economic evaluations because of measurement issues. The results of this study could deviate if presenteeism was not accounted for.

Main strengths of this study were its performance within usual care and the first study we know with active paid employer involvement. Limitations of potential bias due to selection bias and missing data were already addressed. For the calculation of the ICER and ROI the human capital approach was used to calculate productivity losses. The friction cost method assumes workers on sick leave can be replaced, leading to lower productivity losses. The human capital approach, however, resembles the reality of this study better because wages were still paid by employer, while most employees were not (fully) replaced. We applied a linear interpolation assumption for calculating the ICER and ROI. This procedure makes use of educated guesses of linearity for the level of costs per period but was needed because cost variables were not measured every month. Moreover, recall bias may have occurred for medical expenses [45]. Including forgotten medical expenses would result in higher costs. The monetary value per QALY is debated and there is no consented threshold to assess cost-effectiveness [46]. Therefore, multiple threshold values the cost-effectiveness plane were calculated. The data for the study was retrieved using questionnaires, which is a potential source of bias. Moreover, the measurement properties of questionnaires may be suboptimal [26]. In absence of a gold standard, we applied the questionnaires recommended by the Dutch Heath Care Institute. Many data collection limitations of this study could be resolved by applying automatically administered data on absenteeism and cost consumption, however, this will still exclude presenteeism and out-of-pocket costs. Additionally, within the Dutch context it requires that data from different sources become available, which will involve lengthy procedures and high costs to overcome privacy regulations.

## Conclusion

At discharge, VR+ program was cost-effective compared to the VR program. ROI at discharge was positive for employers.

## Footnotes

1 Example: A respondent indicates that he / she has been troubled by 3 working days from health problems and that the work-efficiency / presenteeism score on these days was rated with an 8. One respondent's workday lasts 8 h. The amount of productivity loss is then equal to 3 * (1- (8/10)) * 8 = 0.6 working day or 4.8 h.
